# Correlation of Arterial and Venous pH and Bicarbonate in Patients With Renal Failure

**DOI:** 10.7759/cureus.19519

**Published:** 2021-11-13

**Authors:** Fatima Ayaz, Muhammad Furrukh, Tehreem Arif, Fazal Ur Rahman, Saima Ambreen

**Affiliations:** 1 Department of Medicine, Holy Family Hospital, Rawalpindi, PAK; 2 Department of Nephrology, Holy Family Hospital, Rawalpindi, PAK; 3 Department of Medicine, Rawalpindi Medical University, Rawalpindi, PAK

**Keywords:** arterial blood gas, venous blood gas, ph, bicarbonate, renal failure

## Abstract

Background and objective

Blood gas analysis plays a pivotal role in the management of various respiratory and metabolic disorders. Both arterial and venous samples can be used for blood gas analysis. Arterial blood sampling is technically difficult and is associated with more complications as compared to venous sampling. Many studies have shown the correlation of arterial and venous pH and bicarbonate levels in sepsis, diabetic ketoacidosis (DKA), chronic obstructive pulmonary disease (COPD), and circulatory failure. But, there is a paucity of data, pertaining specifically to the correlation of arterial blood gas (ABG) analysis and venous blood gas (VBG) analysis in patients with renal failure. The objective of this study was to look for any possible correlation between arterial and venous pH and bicarbonate values in patients with renal failure.

Methods

This cross-sectional study was carried out at a large tertiary care hospital in Rawalpindi, Pakistan. Over a period of eight months, 101 patients with renal failure were enrolled after obtaining informed consent. Arterial and venous samples from the patients were obtained, analyzed, and compared.

Results

Out of the total 101 patients, 53 (52.5%) were male while 48 (47.5%) were female. The mean age of the patients was 46.23 ±15.54 years. Mean arterial pH and venous pH were 7.35 and 7.28 respectively. The Pearson correlation coefficient between arterial and venous pH was found to be 0.857 (p<0.001). The mean arterial and venous bicarbonate values were 14.47 mEq/L and 15.51 mEq/L respectively. And the Pearson correlation coefficient between arterial and venous bicarbonate was found to be 0.842 (p<0.001).

Conclusion

Venous pH and bicarbonate levels correlate strongly with arterial pH and bicarbonate levels, respectively, in patients with renal failure.

## Introduction

Blood gas analysis is pivotal in the management of various respiratory and metabolic disorders, as it provides vital information about oxygenation and acid-base status of the body [1]. Both the arterial and venous samples can be used for blood gas analysis. But, arterial blood sampling is technically difficult as compared to venous sampling, often requiring multiple attempts. Arterial sampling is also associated with complications like hematoma formation, infection, arterial thrombosis, or embolization, which can lead to ischemic injury to digits, distally. Moreover, it is a significantly painful procedure causing more discomfort to the patient [2]. On the other hand, venous sampling is easier, less painful, and associated with a lower incidence of side effects [3]. There is a large number of studies that compare the venous and arterial blood gas values. Usually venous blood gas (VBG) has a lower pH and partial pressure of oxygen (pO_2_) but a greater partial pressure of carbon dioxide (pCO_2_) and bicarbonate than the arterial blood gas (ABG). But, it is not clear whether this relationship is either constant or predictable. It is also plausible that disease states affecting venous and arterial flow would result in a greater disparity between the measured variables in the VBG and ABG [2-4].

Although many studies have shown a correlation of arterial and venous pH and bicarbonate levels in sepsis, diabetic ketoacidosis (DKA), chronic obstructive pulmonary disease (COPD), and circulatory failure [5-14], there is still a paucity of data specifically pertaining to the correlation of ABG and VBG in patients with renal failure. A significant number of renal failure patients require blood gas analysis specifically for pH and bicarbonate estimation. In light of this, we designed this study with an objective to look for any possible correlation between arterial and venous pH and bicarbonate values in patients with renal failure, as any possible correlation between these can lead to the substitution of more invasive and technically difficult arterial sampling with the easier and less invasive venous sampling for blood gas analysis.

## Materials and methods

This comparative study was carried out at the nephrology department of Holy Family Hospital, Rawalpindi, Pakistan from January 2020 to August 2020.

Inclusion and exclusion criteria

Patients with renal failure (both chronic and acute) undergoing hemodialysis were included in the study after obtaining informed verbal consent. Patients with concomitant sepsis, COPD, circulatory failure, or DKA were excluded from the study. Patients who refused to participate in the study were also not included.

Ethical approval

Prior to commencement, ethical approval was obtained from the Research and Ethical Committee of Rawalpindi Medical University, Pakistan (Holy Family Hospital is allied with Rawalpindi Medical University). The reference number of the approval letter is R-62/RMU.

Sampling

After obtaining informed consent, 101 patients were included in the study via non-probability convenience sampling.

Data collection procedure and analysis

After receiving informed verbal consent from the patients, relevant details like age, gender, and duration of hemodialysis were noted in proformas. Arterial and venous samples were collected with minimal delay (less than five minutes). Samples were then sent to the lab immediately and were analyzed within 30 minutes by EasyBloodGas^TM^ analyzer (Medica Corporation, Bedford, MA). Complete data were entered into and then analyzed using IBM SPSS Statistics for Windows, Version 25.0 (IBM Corp, Armonk, NY). Means and standard deviations were calculated for quantitative data like age, duration of hemodialysis, pH, and bicarbonate values. Frequencies and percentages were computed for qualitative data. The Pearson correlation coefficient was calculated for arterial and venous values of pH and bicarbonate.

## Results

Of the total 101 patients, 53 (52.5%) were male while 48 (47.5%) were female. The mean age of the patients was 46.23 ±15.54 years. The average duration for which patients were receiving hemodialysis was 278.44 ±593.24 days. The mean arterial and venous pH were found to be 7.35 and 7.28 respectively. The range of arterial pH observed was from 6.90 to 7.64, while the range of venous pH varied from 6.6 to 7.5. The mean arterial and venous bicarbonate levels were 14.47 mEq/L and 15.51 mEq/L respectively (Table 1).

**Table 1:** Comparison of arterial and venous pH and bicarbonate values *Bicarbonate in mEq/L SD: standard deviation

	Arterial, mean ±SD	Venous, mean ±SD	Difference, mean ±SD
pH	7.3553 ±0.12	7.28 ±0.14	0.06 ±0.07
Bicarbonate*	14.47 ±6.67	15.51 ±6.96	-1.04 ±3.84

The Pearson correlation coefficient between arterial and venous pH was found to be 0.857, while the Pearson correlation coefficient between arterial and venous bicarbonate was found to be 0.842. Arterial and venous pH and bicarbonate values were highly correlated (p<0.001).

## Discussion

ABG analysis is the standard method for acid-base assessment. However, the arterial puncture is associated with more side effects and requires an additional vascular puncture apart from routine venous puncture, thereby exposing staff to increased risk of needlestick injury. A number of studies have shown that VBG can be a good alternative to ABG in settings of DKA, mechanical ventilation, COPD, and other acute illnesses [1-6].

Overall, there is a paucity of data specifically pertaining to the comparison of ABGs and VBGs in patients with renal failure. However, many studies have shown a good correlation of arterial and venous pH and bicarbonate levels in patients with DKA, COPD, acute illness, and mechanical ventilation [1,2,4-7]. Our study showed a high level of correlation of pH (r=0.857) and bicarbonate (r=0.842) in arterial and venous blood gas samples. Gokel et al. have shown that in healthy controls, the correlation between arterial and venous pH values (r=0.76.) was moderate but in patients with uremic acidosis, this correlation was high (r=0.98) [9]. A study conducted by Brandenburg and Dire on patients with DKA revealed a high correlation between arterial and venous pH (r=0.9689) and bicarbonate values (r=0.9543) [6]. Another similar study on emergency department patients by Kelly et al. showed a high correlation between arterial and venous pH (r=0.92) [1]. Ak et al. have shown a high correlation between arterial and venous pH (r=0.934) and bicarbonate (r=0.927) in patients with COPD [14].

Limitations of the study include the use of the non-probability sampling technique and the fact that the data were collected from a single center only. Although the patients were enrolled via non-probability convenience sampling, it is unlikely to have resulted in systemic bias. Furthermore, partial pressures of oxygen and carbon dioxide were not included in the study due to the fact that these parameters start changing if samples are not analyzed within 15 minutes and the cut-off limit for the time from sampling to analysis in our study was 30 minutes as mentioned earlier [15].

## Conclusions

Based on our findings, venous pH and bicarbonate levels correlate strongly with arterial pH and bicarbonate levels, respectively, in patients with renal failure. Therefore, VBG analysis can be a reasonably good alternative to ABG analysis in such patients.
